# Supplementary material for: Divergent acyl carrier protein decouples mitochondrial Fe-S cluster biogenesis from fatty acid synthesis in malaria parasites
Source: eLife. 2021 Oct 6;10:e71636. doi: 10.7554/eLife.71636 (PMC8547962; doi:10.7554/eLife.71636)
Supplement: Figure 1—figure supplement 1—source data 1. [file elife-71636-fig1-figsupp1-data1.docx]

| **FASII**  **proteins** | ***E. coli*** | ***P. falciparum***  **apicoplast** | **E-value** | ***P. falciparum***  **mitochondrion** | **E-value** |
| --- | --- | --- | --- | --- | --- |
| acyl carrier protein | AcpP  P0A6A8 | ACP  PF3D7_0208500 | 5e-22 | ACP  PF3D7_1208300 | 3e-09 |
| phosphopantetheine transferase | AcpS  P24224 | ACPS  PF3D7_0420200 | 8e-15 | - | - |
| acetyl-CoA carboxylase | AccC  P24182 | ACC  PF3D7_1469600 | 7e-58 | - | - |
| malonyl-CoA:ACP transferase | FabD  P0AAI9 | MCAT/FabD  PF3D7_1312000 | 4e-39 | - | - |
| 3-ketoacyl-ACP synthase | FabB/FabF  P0A953 | FabB/FabF  PF3D7_0626300 | 1e-48 | - | - |
| 3-ketoacyl-ACP reductase | FabG  P0AEK2 | FabG  PF3D7_0922900 | 4e-80 | - | - |
| 3-hydroxyacyl-ACP dehydratase | FabZ  P0A6Q6 | FabZ  PF3D7_1323000 | 3e-39 | - | - |
| 2-enoyl-ACP reductase | FabI  P0AEK4 | FabI  PF3D7_0615100 | 4e-19 | - | - |
| beta-ketoacyl-ACP synthase III | FabH  P0A6R0 | FabH  PF3D7_0211400 | 9e-68 | - | - |
