# Supplementary material for: Divergent acyl carrier protein decouples mitochondrial Fe-S cluster biogenesis from fatty acid synthesis in malaria parasites
Source: eLife. 2021 Oct 6;10:e71636. doi: 10.7554/eLife.71636 (PMC8547962; doi:10.7554/eLife.71636)
Supplement: Figure 2—figure supplement 1—source data 1. [file elife-71636-fig2-figsupp1-data1.docx]

| **LYR Protein**  **Human/Yeast** | ***P. falciparum***  **homolog** | **E-value** |
| --- | --- | --- |
| LYRM4/ISD11 | Isd11  PF3D7_1311000 | 3e-07/3e-06 |
| LYRM1 | - | - |
| LYRM2 | - | - |
| LYRM3 | - | - |
| LYRM5 | - | - |
| LYRM6 | - | - |
| LYRM7/MZM1 | - | - |
| LYRM8/Sdh6 | - | - |
| LYRM9 | - | - |
| ACN9/Sdh7 | - | - |
| C7orf55/FMC1 | - | - |
| L0R8F8 | - | - |
