# Supplementary material for: Divergent acyl carrier protein decouples mitochondrial Fe-S cluster biogenesis from fatty acid synthesis in malaria parasites
Source: eLife. 2021 Oct 6;10:e71636. doi: 10.7554/eLife.71636 (PMC8547962; doi:10.7554/eLife.71636)
Supplement: Figure 2—figure supplement 5—source data 1. [file elife-71636-fig2-figsupp5-data1.docx]

|  | **ACP bait spectral counts** | | |
| --- | --- | --- | --- |
|  | Exp. 1 | Exp. 2 | Exp. 3 |
|  | Triton | Triton | Digitonin |
| IP: mACP | 73 | 37 | 19 |
| IP: aACP | 63 | 65 | 12 |
