# Supplementary material for: Divergent acyl carrier protein decouples mitochondrial Fe-S cluster biogenesis from fatty acid synthesis in malaria parasites
Source: eLife. 2021 Oct 6;10:e71636. doi: 10.7554/eLife.71636 (PMC8547962; doi:10.7554/eLife.71636)
Supplement: Supplementary file 1. [file elife-71636-supp1.docx]

| **No.** | **Primer Name** | **Sequence (5’ to 3’)** |
| --- | --- | --- |
| 1 | mACP-HA_2_-TEOE f | ACGATTTTTTCTCGAGATGAGAAGAAATATTATTCAAAAAATTCTGTTCAATAATA |
| 2 | mACP-HA_2_-TEOE r | CGTATGGGTACCTAGGTGTTTTTTTTATATTTAATTGAATTATATAATCTATAACT |
| 3 | Rieske-HA_2_-TEOE f | CATTTCGAATAAACACGATTTTTTCTCGAGATGAATAATATTAAATATGTGGAACTTTTTTAC |
| 4 | Rieske-HA_2_-TEOE r | GTAATCTGGAACATCGTATGGGTACCTAGGTCCAATTTTTATCGTATTTTCATCAACAAATTC |
| 5 | Cyt c_1_-GFP-TEOE f | ACGATTTTTTCTCGAGATGGCTGGTGGGGGAG |
| 6 | Cyt c_1_-GFP-TEOE r | CTGCACCTGGCCTAGGTAAATATTTGATTTTTCCAAAGTCAATTCTTCTAGTTG |
| 7 | Nfs1-HA_2_-pET28a f | GTTTAACTTTAAGAAGGAGATATACCATGGCCAAATTTCTTCAAATAATAAAACATCTCAAATTACAAAACAAAAAGAACGCAC |
| 8 | Nfs1-HA_2_-pET28a r | GATCTCAGTGGTGGTGGTGGTGGTGCTCGAGTGTCCATATAAACTTTGGAATGTCGTCATTCGAGGG |
| 9 | mACP-HA_2_-pET28a f | AGGAGATATACCATGGCCAGAAGAAATATTATTCAAAAAATTCTGTTCAATAATAAAAATATAACACTATGTAATACG |
| 10 | mACP-HA_2_-pET28a r | GGTGGTGGTGCTCGAGTTACGCGTAATCAGGTACATCGTATGGATAAGCGTAATC |
| 11 | TrmACP-HA_2_-pET28a f | AACTTTAAGAAGGAGATATACCATGGCCTTGAGCGCTCAAAAAAATAGTGCCTTTTTTTC |
| 12 | TrmACP-HA_2_-pET28a r | ATCTCAGTGGTGGTGGTGGTGGTGCTCGAGTTACGCGTAATCAGGTACATCGTATGGATAAGC |
| 13 | Isd11-His6-pET21d f | GTTAATATTATACAATATACCTAGGATGAATGGGAATC |
| 14 | Isd11-His6-pET21d r | CAGTGAAAAGTTCTTCTCCTTTACTCGTACGTTTGTTAACTAAAGG |
| 15 | mACP­_gRNA_sense | TAAGTATATAATATTTGGTATTGTTATATTAAATTGTTTTAGAGCTAGAA |
| 16 | mACP­_gRNA_antisense | TTCTAGCTCTAAAACAATTTAATATAACAATACCAAATATTATATACTTA |
| 17 | Phe-Ala mACP-HA_2_ f | GGATAATCGAGCCTGGGATGCTCTTGATACCGTTGAATTTTTAATAGATATTG |
| 18 | Phe-Ala mACP-HA_2_ r | CAATATCTATTAAAAATTCAACGGTATCAAGAGCATCCCAGGCTCGATTATCC |
| 19 | LYR-AAA Isd11 f | GTTAATATTATACAATATACCTAGGATGAATGGGAATCAAATAAAACAACTTAAGAAGGCTGCCGCGCATATATTGAATGAAGC |
| 20 | LYR-AAA Isd11 r | GAAGCTTCATTCAATATATGCGCGGCAGCCTTCTTAAG |
| 21 | mACP 5’UTR f | CCATACATATTTGTGTATGTATGTATGTATTTATTTATTATGTATTCAAATTATGC |
| 22 | mACP 3’UTR r | TTCTTCTCATCTTAAGCTATTTTATATTTATAATATAAATGAGCTCTTCAAAAGTAG |
| 23 | 3’ Aptamer tag r | CCCAGGCCTCTAGTTTAC |
| 24 | L12A Isd11 f | GTTTAACTTTAAGAAGGAGATATACCATGGCCAATGGGAATCAAATAAAACAACTTAAGAAGGCTTATCGTCATATATTGAATGAAGCTTC |
| 25 | Y13A Isd11 f | GTTTAACTTTAAGAAGGAGATATACCATGGCCAATGGGAATCAAATAAAACAACTTAAGAAGTTAGCCCGTCATATATTGAATGAAGCTTC |
| 26 | R14A Isd11 f | GTTTAACTTTAAGAAGGAGATATACCATGGCCAATGGGAATCAAATAAAACAACTTAAGAAGTTATATGCACATATATTGAATGAAGCTTC |
| 27 | aACP_F | ACGATTTTTTCTCGAGATGAAGATCTTATTACTTTGTATAATTTTTC |
| 28 | aACP_R | CGTATGGGTACCTAGGTTGCTTATTATTTTTTTCTATATAATCTATAGCATCTTGA |
| 29 | Proc_aACP_F | AGGAGATATACCATGGCCTTAAATAGAAAAAATGATTACAATTTGATAAAAAATAAAAACCCATCTAGCTC |
| 30 | Proc_aACP_R | GGTGGTGGTGCTCGAGTTACGCGTAATCAGGTACATCGTATGGATAAGCGTAATC |

**Supplementary File 1.** PCR primers used for cloning and genotyping.
